# Fiberoptic Intubation Using LMA™ as A Conduit and Cook^®^ Airway Catheter as An Exchanger in A Case of Tessier 7 Facial Cleft Syndrome

**Published:** 2009-04

**Authors:** D Dasgupta, Anand Jain, Vaibhavi Baxi, A Parab, A Budhakar

**Affiliations:** 1 Head of the Department, Department of Anaesthesia, Jaslok Hospital and Reserch Center, Mumbai; 2 DNB student, Department of Anaesthesia, Jaslok Hospital and Reserch Center, Mumbai; 3 DNB student, Department of Anaesthesia, Jaslok Hospital and Reserch Center, Mumbai; 4 Consultant Anaesthetist , Department of Anaesthesia, Jaslok Hospital and Reserch Center, Mumbai; 5 Consultant Anaesthetist , Department of Anaesthesia, Jaslok Hospital and Reserch Center, Mumbai

**Keywords:** Tessier syndrome, Macrostomia, LMA™, Fiberoptic bronchoscope, Cook® airway catheter

## Abstract

**Summary:**

Any anaesthesiologist handling a paediatric airway must have a detailed understanding of the differences in airway anatomy, signs and symptoms of airway compromise and common paediatric airway abnormalities. In addition to various equipments needed to manage a difficult airway, there should be a clear plan for evaluation, preparation and management of life threatening complications. We share our experience of successfully managing a difficult airway of a 5 year old child with Tessier 7 facial cleft syndrome. We emphasize the importance of preoperative evaluation, preparation and use of various airway adjuncts.

## Introduction

In 1976, Tessier proposed an anatomic classification of rare facial, craniofacial, and laterofacial clefts. In this classification, the orbit is used as the primary structure of reference. Fifteen locations for clefts can be differentiated. The classification facilitates an understanding of tridimensional structure of these deformities.^1^ The etiology of these clefts is an embryonic developmental failure of structures derived from 1st & 2nd branchial arches resulting in maxillary, mandibular and aural hypoplasia. The clinical expression of Tessier 7 ranges from a preauricular skin tag to a cleft across the cheek from angle of the mouth to the ear. When the cleft is bilateral, it leads to gross macrostomia.^1^

## Case report

**Fig 1:**
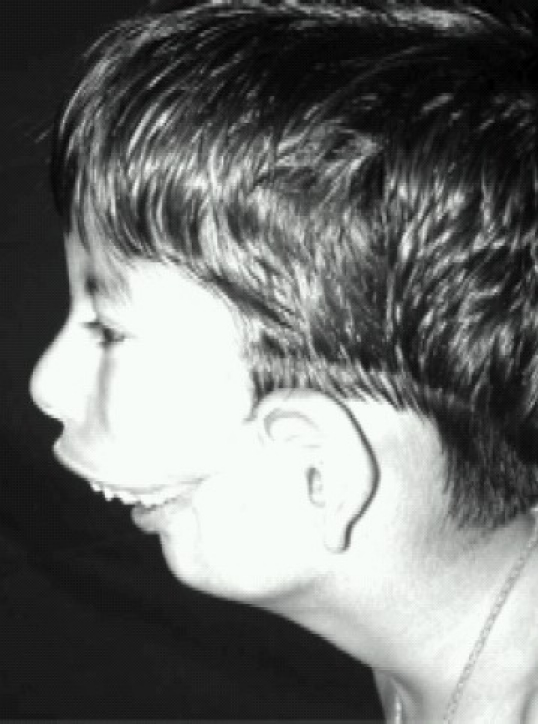
Showing lateral view

**Fig 2:**
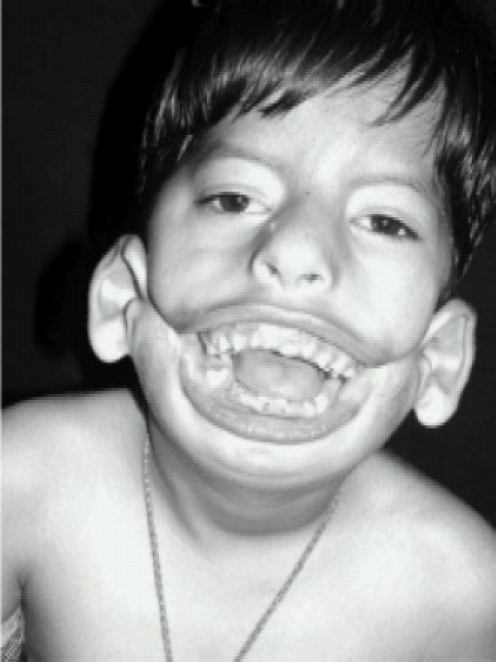
Front view showing mouth opening

A 5-year-old male child, known case of bilateral Tessier 7 facial cleft syndrome, was posted for total correction of facial deformity. He was a preterm child delivered vaginally. Presence of gross macrostomia at birth precluded breastfeeding. Till the age of 3 months, he was fed through a nasogastric tube after which a feeding gastrostomy was done under general anaesthesia, details of which were not available. Feeding gastrostomy was closed at the age of 2 years under GA, the details of which are not known. The child was on liquids and semi solid food since then. Before the surgery at our Hospital, he was kept NBM for 6 hours. There were no other associated congenital deformities. Milestones were normal. A history of snoring and repeated upper respiratory tract infections was present. On general examination, height was 95cm and weight was 15kg with a low set of ears. Airway examination revealed a facial cleft extending from angle of the mouth across the cheek to the ear on both sides amounting to gross macrostomia (Fig 1 & 2). Mouth opening was 3cm (Fig 1 Showing lateral view), Mallampatti classification-IV and thyromental distance-2.5cm with severe retrognathia. Teeth were severely maloccluded. Neck movements and other systems were normal. Routine investigations were normal. Preoperatively, the child was kept fasting for 6 hrs. Venous access was taken in the ward. No sedative premedication was administered in the ward due to history of snoring. In the operation theater, a difficult airway cart including fiberoptic bronchoscope and tracheostomy kit was kept ready. Monitoring included a precordial stethoscope, electrocardiogram, pulse oximeter, non invasive blood pressure cuff, capnogram and temperature probe. Plan of intubation was to secure a non surgical airway in an anaesthetised patient. Awake fiberoptic intubation was not possible since it would require a quite cooperative child for good bronchoscopic views. Our PLAN A was to attempt a direct laryngoscopy after sevoflurane induction. PLAN B was to do LMA™ guided fiberoptic intubation. For preoxygenation, we had to use a No 5 face mask due to the gross macrostomia. Gamgee pads were applied around the mask to reduce the leak and improve the seal. Injection glycopyrrolate 0.06 mg was administered and the child was anaesthetised with sevoflurane. Direct laryngoscopy revealed Cormack Lehane IV. Since it was becoming increasingly difficult to maintain the airway with mask, we decided to introduce an LMA™ size 2½. After confirming adequate ventilation with the LMA™, fentanyl 30 mcg, propofol 30 mg, atracurium 10 mg were given intravenously. Then, through the LMA™, fiberoptic bronchscope with a 5mm safety flex tube mounted on it was introduced. The tube was then passed across the cords to secure the airway.

**Fig 3:**
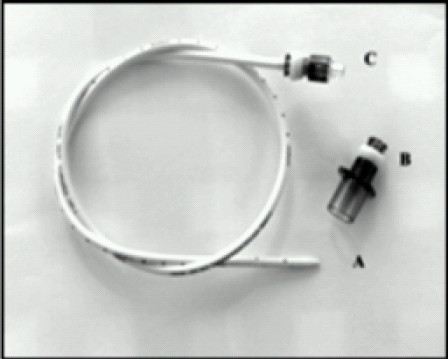
Cook' catheter

Although the child was being ventilated adequately, the LMA™ had to be removed as it would hinder the oral surgery. Hence, we decided to remove the LMA™-tube assembly over a Cook® airway exchange catheter (Fig 3). Finally, the same 5mm safety flex tube was then railroaded back over Cook® catheter and ventilation was confirmed with EtCO2. We have used two uncuffed tubes of the same size without the connectors. The distal tip of the upper tube including the Murphy eye was cut away and then the cut end was plugged into the enlarged cavity of the proximal end of the lower tube to form a stable tube of double length. The tube was then fixed in midline with surgical sutures and the throat was packed. Surgery was uneventful. Post operatively the child was shifted to Paediatric ICU on ventilator.

## Discussion

**Fig 4:**
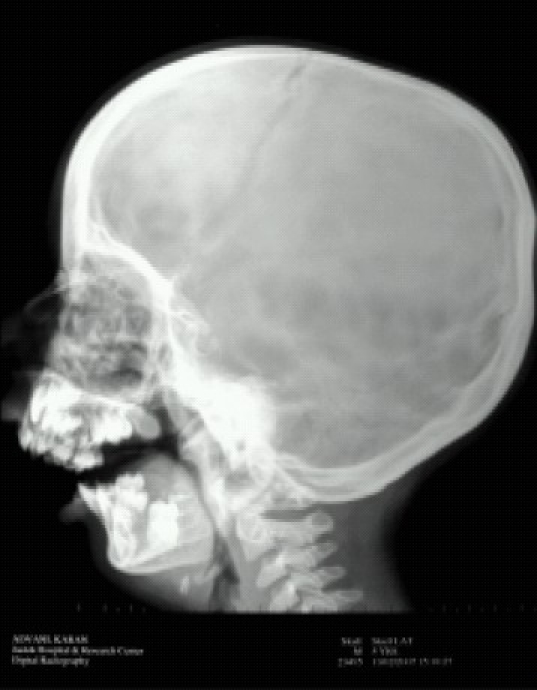
X ray skull lateral view showing malocclusion of teeth

Children with Tessier 7 syndrome often prove to be difficult candidates for airway management. Huge macrostomia leads to a difficult mask fit, while intubation can be very challenging. Always anticipate difficult airway and choose an appropriate technique.^2^ Direct laryngoscopy can either be difficult or impossible. Multiple attempts cause edema and bleeding with subsequent difficult ventilation. (Fig 2 Front view showing mouth opening, Fig 4 X ray skull lateral view showing malocclusion of teeth). Hence an alternative technique to secure the airway without traumatizing the larynx should be sought. In our patient, following direct laryngoscopy, we were unable to visualize even the epiglottis. So we went to our plan B, which was LMA™ guided fiberoptic intubation.^3^ Performing an awake fiberoptic intubation in paediatric population is challenging, if not impossible. For intubation through LMA™, both blind as well as fiberoptic techniques are described. However, blind methods should be avoided due to the risk of trauma and bleeding. LMA™ guided fiberoptic intubation has gained popularity as the LMA™ provides a patent airway, a conduit for the bronchoscope and control ventilation at the same time.^4^,^5^ If the LMA™ does not interfere with the surgery, it can be left in place. However the greatest challenge encountered when intubating through an LMA™ is its removal without dislodging the endotracheal tube. This difficulty is unique to paediatric airway as the lengths of an age appropriate endotracheal tube and LMA™ are similar. The proximal end of the tube tends to disappear into the LMA™ once the tube has passed through the vocal cords.^6^ Various methods have been described to circumvent this problem. Most commonly, this is done by removing the tube connector and attaching another similar sized tube proximally to the first tube.^7^ Peter et al^8^ have described the use of LMA™ guided fiberoptic intubation and Cook® airway exchange catheter in difficult paediatric airway. The advantages of this catheter are long length, atraumatic, continuous access to airway and ability to oxygenate and ventilate during exchange process.

Walburn et al^9^ have used a guidewire for the exchange of LMA™-tube assembly in a difficult paediatric case. However, the guidewire proves to be too soft and thin for support. Use of gum elastic bougie, adult stylets and ureteral dilators have also been described.^10^ One must always anticipate, prepare and plan for a difficult paediatric airway. Various airway adjuncts should be used judiciously. LMA™ guided fiberoptic intubation has become gold standard for difficult paediatric airway. Cook® airway catheter should be used as an exchanger in difficult airway as it has distinct advantage over other adjuncts.
